# Aging progression of human gut microbiota

**DOI:** 10.1186/s12866-019-1616-2

**Published:** 2019-10-28

**Authors:** Congmin Xu, Huaiqiu Zhu, Peng Qiu

**Affiliations:** 1 Department of Biomedical Engineering, College of Engineering, Peking University, Beijing, 100871 China; 2 Department of Biomedical Engineering, Georgia Institute of Technology and Emory University, Atlanta, 30332 USA

**Keywords:** Human gut microbiota, Aging, Sample progression discovery, 16S rRNA sequencing

## Abstract

**Background:**

Human gut microbiota are important for human health and have been regarded as a “forgotten organ”, whose variation is closely linked with various factors, such as host genetics, diet, pathological conditions and external environment. The diversity of human gut microbiota has been correlated with aging, which was characterized by different abundance of bacteria in various age groups. In the literature, most of the previous studies of age-related gut microbiota changes focused on individual species in the gut community with supervised methods. Here, we aimed to examine the underlying aging progression of the human gut microbial community from an unsupervised perspective.

**Results:**

We obtained raw 16S rRNA sequencing data of subjects ranging from newborns to centenarians from a previous study, and summarized the data into a relative abundance matrix of genera in all the samples. Without using the age information of samples, we applied an unsupervised algorithm to recapitulate the underlying aging progression of microbial community from hosts in different age groups and identify genera associated to this progression. Literature review of these identified genera indicated that for individuals with advanced ages, some beneficial genera are lost while some genera related with inflammation and cancer increase.

**Conclusions:**

The multivariate unsupervised analysis here revealed the existence of a continuous aging progression of human gut microbiota along with the host aging process. The identified genera associated to this aging process are meaningful for designing probiotics to maintain the gut microbiota to resemble a young age, which hopefully will lead to positive impact on human health, especially for individuals in advanced age groups.

## Background

The human gut, as an eco-system embodying more than 100 trillion microbes, plays an important role in human health [1]. The structure and composition of the gut flora are the result of long-term natural selection acting on both the microbes and host, which finally promotes mutual cooperation and functional stability of this complex ecosystem [2]. Factors such as diet, environment, host genetics and pathological conditions are important factors for explaining the variation of gut microbial community in different individuals [3–7]. Aging process captures many facets of biological variation of the human body, which leads to functional decline and increased incidence of infection in gut of elderly people [8]. Age-related changes of human gut microbiota have been revealed by several studies [9–17]. Hopkins et al. found higher numbers of *Enterobacteria* in children’s fecal than adults through culturing microbes [9]. Using 16S rRNA sequencing, Yatsunenko et al. found *Bifidobacterium* declined with increasing ages [11]. Odamaki et al. revealed that aging was accompanied by increasing proportion of *Bacteroides*, *Eubacterium* and *Clostridiaceae*; *Enterobacteriaceae* were enriched in infant and elderly; *Bifidobacterium* were enriched in infants; *Lachnospiraceae* were enriched in adults [10]. Using whole genome sequencing, Stewart et al. discovered decline of L-lactate dehydrogenase (milk fermentation) and increase of transketolase (metabolism of fiber) over the first year of life [13]. In these studies, various supervised machine learning methods have been applied, including multi-group comparative analysis with permutational analysis of variance (PERMANOVA) [9, 10, 12, 17], Spearman rank correlation and Random Forest [11], as well as frequency-inverse document frequency and minimum-redundancy maximum-relevance [14], which effectively identified taxonomic or functional signatures showed aging-related changes of gut microbiota.

In this study, we proposed to explore an unsupervised machine learning approach for identifying aging-related progression of microbiota community and bacteria genera associated with the progression. The unsupervised algorithm adopted here is called Sample Progression Discovery (SPD), which was developed to identify progressive changing patterns of gene expression that reflect the biological progression in various biological processes and systems [18]. This idea was first applied to microarray gene expression analysis [18], and then extended to flow cytometry [19] and single-cell RNA-seq analysis [20]. Here, we applied SPD on community profiles extracted from 16S rRNA sequencing data of human gut microbiota samples in various age periods ranging from new-born babies to centenarians. SPD recapitulated the underlying aging progression in the data in an unsupervised fashion, and sorted the gut microbiota samples in an order consistent to the host ages. In addition, SPD identified bacteria genera associated with the aging-related progression of gut microbiota. These findings demonstrated the existence of an aging progression of human gut microbial community, and points to important bacteria genera that characterize the aging of gut microbiota.

## Results

### Data annotation and samples overview

We obtained a total of 3.2 million high-quality 16S rRNA sequences from 368 samples [10], with 8734±2748 (mean ± deviation) reads per sample. The 16S rRNA sequences were binned into 366 genera using the Mothur pipeline [21] with SILVA [22] as reference database (see Methods). We removed 119 genera with extremely low abundance, the total amount of sequences annotated as these genera only accounted for 0.01% of all the sequences. Also, we excluded one sample with abnormally high proportion of *Pseudomonas*, which is an indication of abnormal sampling or pathological disorder of this individual ‘Japanese 320’. Overall, we derived a relative abundance matrix of the 247 genera across the 367 samples, which served as the basis for further analyses. To reveal age-related progression of gut microbiota, we divided the samples into 14 age groups considering body transformation periods. New-born babies were grouped according to their weaning status and adults were grouped by decade (Table 1). Except the centenarians, there were at least 10 samples in each age group.

**Table 1:** Samples were grouped into 14 age-segment groups

Group	Age segmentation	Number of samples	Female	Male
1	(0, 0.4]	10	6	4
2	(0.4, 1.2]	12	4	8
3	(1.2, 3]	19	9	10
4	(3, 9]	14	8	6
5	(9, 19]	10	3	7
6	(19, 29]	40	24	16
7	(29, 39]	88	43	45
8	(39, 49]	34	21	13
9	(49, 59]	25	13	12
10	(59, 69]	28	17	11
11	(69, 79]	15	10	5
12	(79, 89]	48	32	16
13	(89, 99]	19	15	4
14	≥100	5	5	0

The first three groups of new-born babies were classified regarding their weaning status, i.e. before weaning, weaning and after weaning separately. Other samples were grouped by decade

**Fig. 1:**
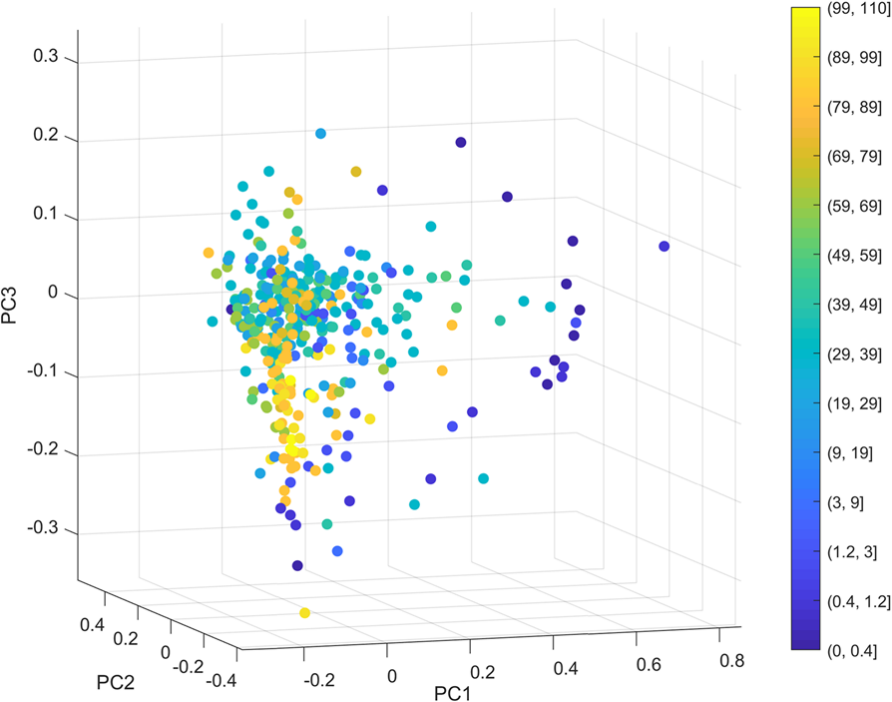
Sample overview using PCA. Using the relative abundance of 247 genera across all the 367 samples as input, we linearly transformed and visualized the data in a three-dimensional space. Each sample is represented by one dot, colored according to age. Samples from children younger than three (the dark blue dots) scattered most distantly, while older age groups were mixed together in the PCA space

We performed PCA to visualize the taxonomic patterns of these samples in a low-dimension space based on the relative abundance matrix of the 247 genera across the 367 samples. The top three principle components explained 33.17%, 15.09% and 10.32% of the original data variance, respectively. As shown in Fig. 1, the samples from children younger than three years old scattered loosely, and were quite different from each other. This observation was consistent with previous literature [11], which concluded that intrapersonal variation decreased as a function of age. Nevertheless, the samples did not form distinct groups when visualized by this linear approach.

### Age-related variation of gut microbiota revealed by supervised methods

We applied two previous statistical approaches to identify the age-related variation of the gut microbiota in a univariate fashion. First, we applied permutational one-way ANOVA test [23] to the genus relative abundance matrix to identify genera that significantly varied in different age groups. The abundances of 43 genera showed significant difference across the age groups with *P*<0.001 (1000 randomizations), and the *P* values were adjusted using Bonferroni correction (see more details in Additional file 1). We also applied Spearman correlation to find genera that co-vary with age. There were 17 genera positively correlated with aging and one genus negatively correlated with aging (Additional file 2). These results were consistent with multiple previous literatures, showing that individual genus in the gut microbial community varied during the host aging [9, 10, 12, 17]. Further question naturally arose as to whether the gut microbial community as a whole shift continuously during aging.

### Aging progression of gut microbiota revealed by unsupervised analysis

**Fig. 2:**
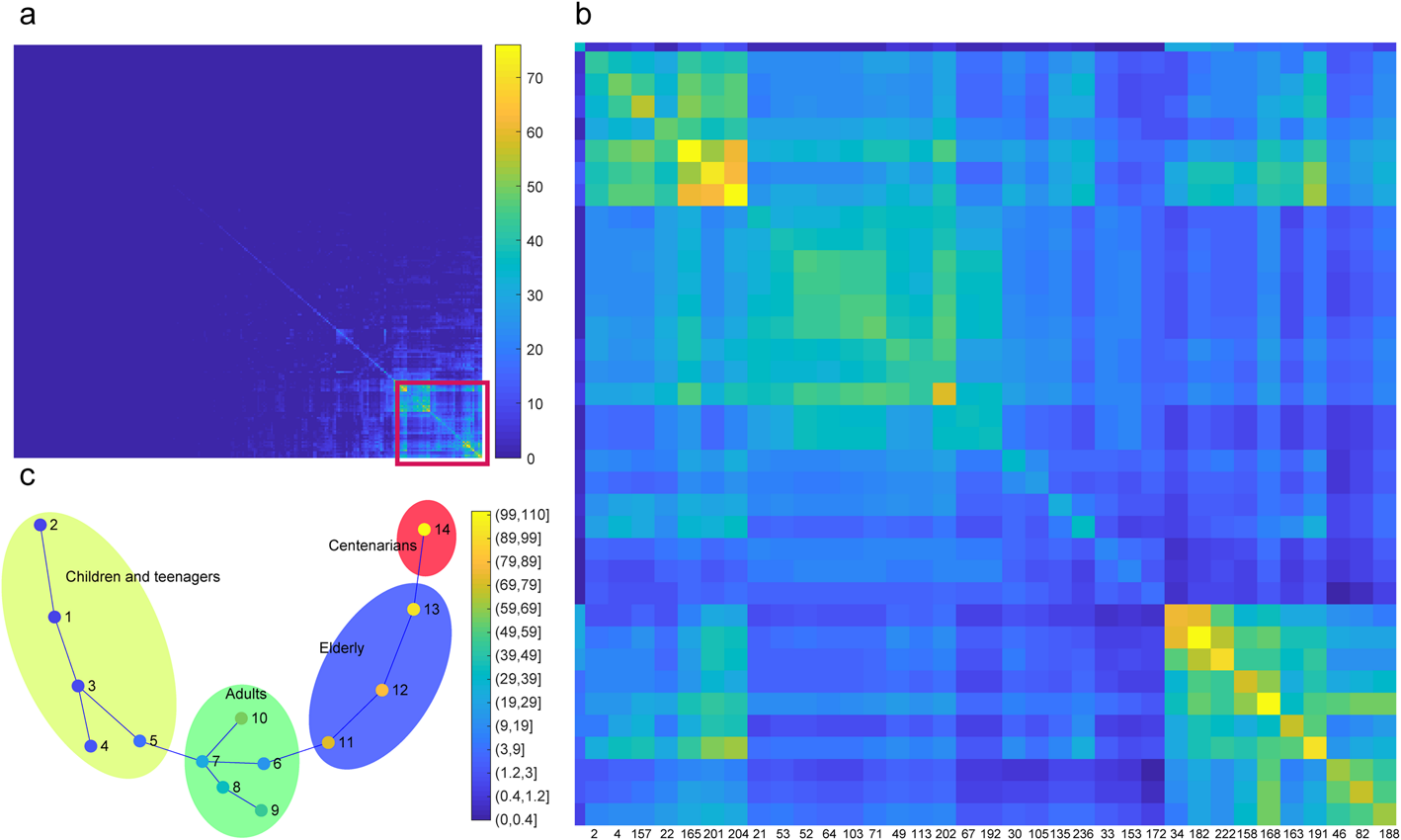
SPD recovered aging progression with taxonomical composition of human gut microbiota. **a** Progression similarity matrix for all genera, with each element counting the number of progression orderings the two corresponding genera shared. **b** We manually picked the highlighted area from (**a**). These selected genera were consistent with a common set of putative progression orderings. **c** An overall minimal spanning tree of the 14 age groups based on the selected genera. Each node represents one age group

Different from the previous supervised univariate methods searching for features that co-varied with aging, we applied an unsupervised method SPD to examine the gut microbiota data in a multivariate fashion. The input to SPD was the averages of genus relative abundance of samples in each age group, which is a 247×14 matrix. The relative abundance of each feature was normalized across samples to release the scale effect. Based on each of the genus features, a minimum spanning tree (MST) was constructed according to Euclidean distance, which represented a putative progression ordering among the 14 sample groups. The 247 genera and the 247 resulting MSTs were cross compared to examine whether multiple genera fitted well the same progression ordering among the samples. Results of these comparisons were summarized into a progression similarity matrix, where each element counted the number of progression orderings that two genera both fit well with. As shown in Fig. 2a and magnified in Fig. 2b, the progression similarity matrix revealed a subset of 35 genera (Additional file 3) that fitted well with a common set of putative progression orderings. Using this subset of genera, an overall minimal spanning tree was constructed to represent the common progression ordering, shown in Fig. 2c. Each node of the tree represented one age group. Nodes were labeled and colored according to their age groups to assist the visualization. However, the age information was not used to determine the structure of the tree. This overall minimal spanning tree is what SPD aimed to identify, a progression ordering among the samples, with respect to which multiple features exhibited gradual changes. The overall minimal spanning in Fig. 2c recapitulated the age progression ordering across the 14 sample groups. Especially, when we further classify these sample groups into four larger groups, i.e. Children and teenagers, Adults, Elderly and Centenarians, the order of sample groups on this minimal spanning tree perfectly matched with the ages of sample groups. This is an interesting result, because SPD was able to recover the correct ordering of aging progression based on the genus relative abundance alone, which implied that there existed an aging progression of the human gut microbiota.

### critical genera underlying the aging progression of gut microbiota

We further examined the 35 selected genera that contributed to the aging progression of gut microbiota, and compared to previous methods. 11 of the 35 genera were significant in the permutational one-way ANOVA analysis with adjusted *P*<0.001. Among the remaining 24 genera detected by SPD only, a few were previously implicated in the literature, such as *Oxalobacter*, *Butyrivibrio*, *Lactobacillus* which have been experimentally demonstrated to be associated with aging [24–26], as well as *Prevotellaceae* which has been highlighted with lower presence in the gut microbiota of centenarians [27]. Among the 35 genera selected according to the progression similarity defined by SPD, only 9 exhibited monotonic changes with respect to aging, while the rest first increased and then descreased in different age periods (Additional file 4: figure 1). This was because SPD was designed to identify features that exhibited gradual changes with respect to a common underlying progression pattern, and the gradual changes were not limited to be monotonic. Therefore, this analysis was able to identify genera that gradually changed without abrupt fluctuations during aging. We performed extensive literature review of these 35 genera, and found a lot of previous reports of the functional relevance of these genera.

**Fig. 3:**
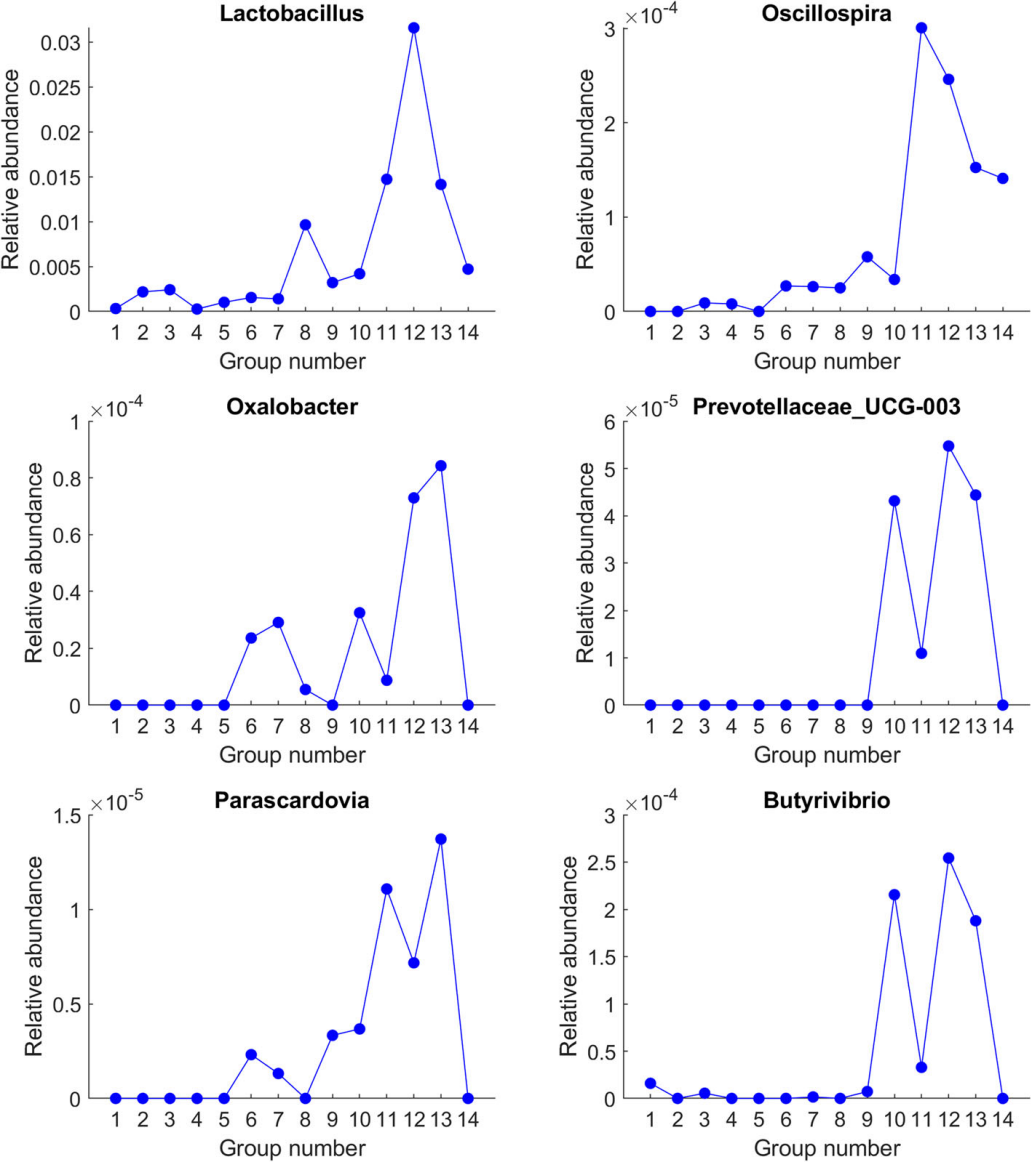
Genera that first increased and then decreased during aging, especially sharply decreased in the 13th or 14th age groups, or both

Genera shown in Fig. 3 shared one common feature. Their abundances increased with respect to aging, but decreased in the extremely elderly subjects. Among these genera, *Lactobacillus* species are commonly used as probiotics [28]. *Oscillospira* species have been frequently reported as enriched in lean subjects compared to the obese subjects [29–32], and are central to the human gut microbiota for degrading fibers [33]. *Oxalobacter* is responsible for degrading oxalate in the gut. It has been experimentally demonstrated appearing in the gut of almost all young individuals, but these bacterium may later be lost during aging [24]. *Prevotellaceae* is commonly found in the gastric system of people who maintain a diet low in animal fats and high in carbohydrates [34] and is lost in centenarians [27]. Researchers also found that there was an increased abundance of *Prevotellaceae* in the guts of healthy people compared with people with Parkinson’s disease [35]. *Parascardovia* is a genus of *Bifidobacteriaceae*, which has been shown to provide health-promoting benefits to the host [36]. *Butyrivibrio* species have been experimentally proved as butyrate producing bacteria, and butyrate is a preferred energy source for colonic epithelial cells and is thought to play an important role in maintaining colonic health in humans [37]. Overall, the decrease of these beneficial genera in the elderly age groups, especially centenarians, maybe manifestation of or causial associations to decline of health in those age groups.

**Fig. 4:**
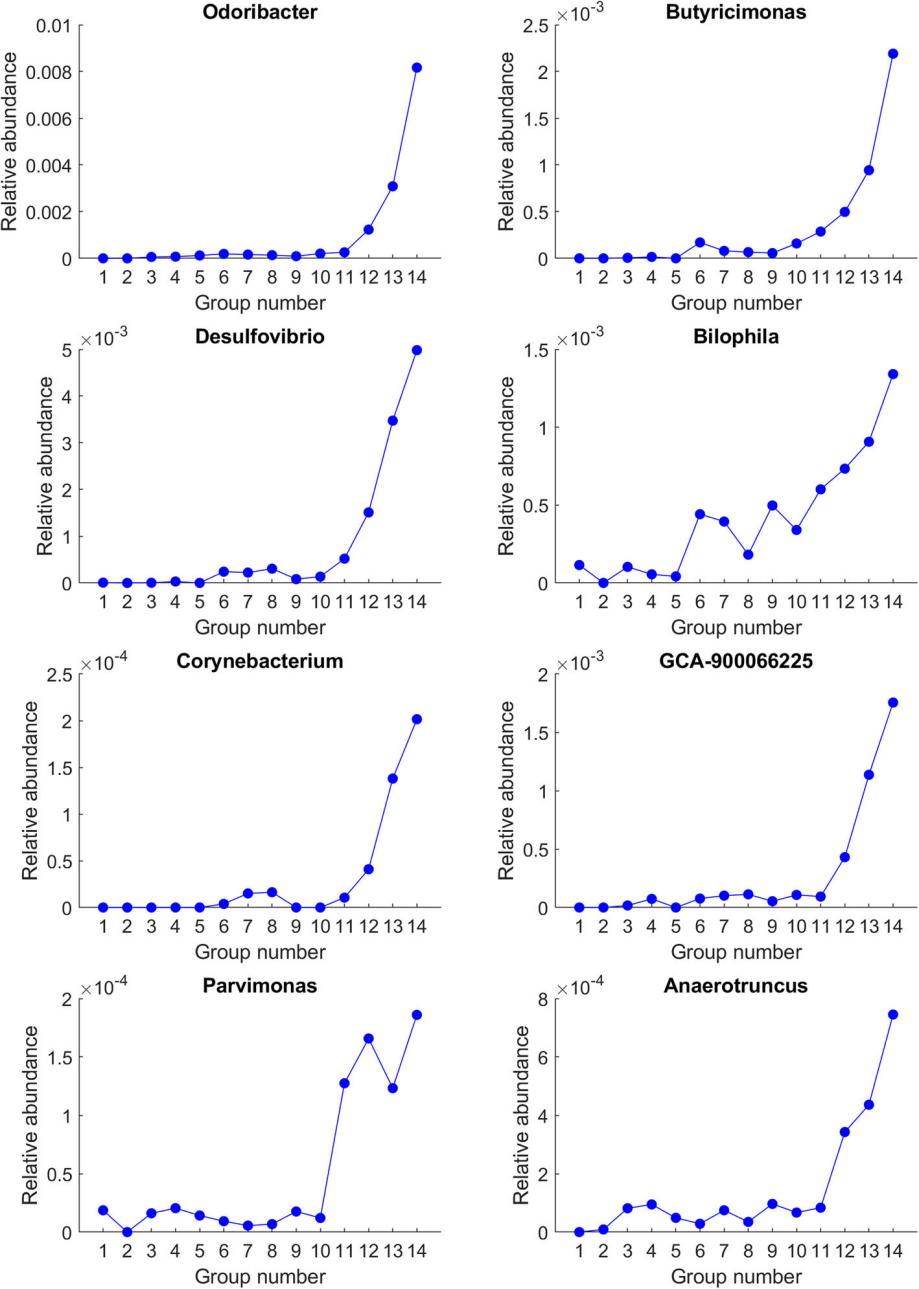
Genera that exhibited general increasing patterns during aging

In contrast, genera in Fig. 4 showed generally monotonically increasing patterns with respect to aging. *Parvimonas* has been reported to be enriched in colorectal cancer [38–42]. *Anaerotruncus* was relatively enriched in patients with age-related macular degeneration [43]. *Corynebacterium* was reported as more abundant in the gut of autistic individuals (autism spectrum disorders) [44]. Many *Corynebacterium* species were also reported as involved in human and animal diseases [45]. GCA-900066225 is one genus in the *Lachnospiraceae* family, which has been reported to be associated with ulcerative colitis, Crohn’s and celiac disease, as well as the stress of the host [46]. *Desulfovibrio* species produce hydrogen sulfide using sulfate as the electron acceptor, and these sulfate-reducing bacteria are positively associated with inflammation [47, 48]. A human stool-derived *Bilophila wadsworthia* strain caused systemic inflammation in specific-pathogen-free mice [49]. Tumor-bearing mice showed enrichment in species of *Odoribacter* [50]. *Butyricimonas* was enriched in the subjects suffering from high rectal temperature, systolic blood pressure, and heart rate and a significantly lower physical activity score [51]. Overall, these monotonically increasing genera were often linked to inflammation and diseases.

All these prior literature of the identified genera pointed to one interesting observation. Many of the genera that first increased and then descreased were previously implicated as health beneficial, whereas most of the monotonically increasing genera were frequently reported as disease-related. When individuals turn elderly beyond 90s, their guts tend to lose some of the beneficial genera and gain potentially harmful genera.

## Discussion

Since the variation of gut microbiota is closely linked to the health status of the host body, an ideal dataset for studying aging of human gut microbiota should be collected from healthy subjects of various age groups. Unfortunately, the health status of individuals included in this study is unknown, because that the data were obtained from a published paper [10] which did not provide the health information corresponding to the samples. During our literature search on age-related changes of the human gut microbiota, we found in multiple previously published papers [10, 11, 14, 52, 53] that the health status of individuals in such studies are often not reported.

In order to gauge the health status of samples in the dataset used in this paper, we performed additional analysis by referencing to multiple previous datasets on the human gut microbiome of hosts suffering from different diseases [4, 5, 7, 54–57]. In each of the previous datasets, we obtained the relative abundance of the human gut microbial genera, and visualized their distributions for both healthy controls and disease samples.. Multiple genera were significantly enriched in the diseases compared to the healthy controls. Most of these genera have been reported as opportunistic pathogens of the human gut [58–67]. These disease-enriched genera typically showed higher abundance and higher variance in disease subjects compared to the healthy ones (first and second columns of Additional file 4: figure 2), while all of these genera exhibit low abundance in the dataset used here (third column of Additional file 4: figure 2). This observation indicated that the samples in the current dataset are more similar to the healthy samples in the previous datasets, and dissimilar to the diseases samples. This comparison demonstrated that majority of the samples in this dataset were derived from healthy subjects.

For 16S rRNA sequencing data analysis, OTU (operational taxonomic unit) is another commonly used classification unit, which allows for binning sequences into features at finer resolution compared to the genus level features. To confirm the observations in our genus level analysis, we applied the progression analysis to the OTU level features. 4663 OTUs were defined by clustering sequences with similarity threshold set as 0.97 for species level classification. After filtering out OTUs with extremely low abundances, the averages of the relative abundances of the remaining 1229 OTUs were calculated for each age group. Progression analysis based on OTU features was able to partially recapitulate the correct order of the age groups (Additional file 4: figure 3), but slightly worse compared to the result from genus level analysis shown in Fig. 2c. It is reassuring that the progression analysis at both OTU level and genus level consistently revealed aging related progression of the human gut microbiota.

In the metagenomics literature, the alpha diversity and the beta diversity are popular metrics for providing quantitative summaries of species diversity. We computed the alpha diversity and the beta diversity based on the averages of genus relative abundance of samples in each age group. The alpha diversity was quantified by the Shannon index and the beta diversity was quantified by Bray-Curtis dissimilarity between different age groups. Additional file 4: figure 4 shows the alpha diversity computed for each individual age group, which showed a steady increase of the alpha diversity as a function of aging, except for the steep drop in the extremely elderly age group [99, 110]. This is consistent with the results shown in Fig. 3, where multiple aging-related genera showed significant decrease in the extremely elderly age group. The beta diversity quantified the dissimilarity between different age groups (Additional file 4: figure 5). Focusing on the beta diversity between neighboring age groups, we observed that the dissimilarity between groups [2, 3] and between groups [13, 14] were notably larger than the dissimilarity between other neighboring age groups. The distinction between group 2 (weaning) and group 3 (weaned) is mainly due to the transformation of weaning status, which is accompanied by drastic dietary changes. However, samples of group 13 and group 14 are all elderly individuals with continuous ages, and the large dissimilarity between groups 13 and 14 cannot be explained by changes of dietary habits. Therefore, we conjecture that the large dissimilarity between groups 13 and 14 is due to the aging of gut microbiota, manifested in the sudden decrease of multiple genera in the extremely elderly samples. Overall, according to both alpha and beta diversity, we can see indications consistent to our observation of the sudden decrease of multiple genera in extremely elderly age samples shown in Fig. 3.

## Conclusions

We applied an unsupervised machine learning approach SPD on genera abundance profile of human gut microbiota quantified by 16S rRNA sequencing data. Without using the age information of the samples, SPD sorted sample groups on a minimal spanning tree that recapitulated the aging progression. This result indicated the existence of an aging progression reflected in the human gut microbiota. In the meantime, we found 35 genera associated with this age-related progression. Some of these genera were not identified using the commonly-used statistical approaches for metagenomics analysis. Literature review of these 35 genera led to a lot of evidences of the functional relevance of these genera. The evidences collectively indicated an age-related decline of the beneficial functions of gut microbiota, as well as increase of inflammation and diseases, especially for the elderly people older than 90s.

## Methods

### Data and data annotation

Our study includes 371 samples of subjects ranging from new-born babies to centenarians, which have been described in publication [10]. We downloaded the 16S rRNA data from DNA data bank of Japan with accession number DRA004160. Three samples were discarded because of only one end of paired-end reads were released. We performed 16S rRNA data processing using Mothur [21]. Low quality reads with average quality score <25 or read length <150bp were filtered out. We set the minimum length of reads as 150bp because the overlap region of each pair of reads was about 150bp. The number of reads in each sample was Gaussian distributed (8734±2748), which implied that all the 368 samples were sequenced in normal depth. The high-quality reads with both paired ends were merged as sequences. Those low-quality reads or reads with only one end were discarded. Then we aligned the sequences against Silva reference database version 132 [22] to infer taxonomical composition of samples. Threshold for the alignment was set as bootstrap confidence value 80% (80% identity) during 100 iterations. Based on the alignment result, we revealed the taxonomic composition at genus level. There were 368 genera in all the samples.

### Feature matrix

We defined the genus abundance matrix N = {n _*ij*_}, where n _*ij*_ is the number of reads of sample *i* binned into genus *j*. One hundred nineteen genera were filtered out for their extremely low abundance, and three genera were combined into one genus cluster as “unclassified”, after which 247 features were obtained for further analysis. To normalize out the variation in sequencing depth of different samples, the genus abundance matrix was transferred into a relative abundance matrix F = {f _*ij*_}, where f _*ij*_=$n_{ij}/\sum _{k=1}^{247}n_{i,k}$. One sample from subject “Japanese 320” was discarded for its abnormally high proportion of *Pseudomonas*. Finally, we have a 367×247 relative abundance matrix *F* for further analysis.

With decent numbers of observations in different age periods, we estimated the genus relative abundance of population in each age group by calculating the mean value of samples in corresponding group, which partially reduced the variations across individual samples and sparsity of the data matrix. Age segments were defined concerning the physiological transition of the host bodies, wherein the new-born babies were grouped according to their weaning status and the adults were grouped by decade. The number of samples in each age group was depicted in Table 1.

## Supplementary information

**Additional file 1** Genera significantly varied in different age groups.

**Additional file 2** Genera correlated with ages revealed by Spearman correlation. Validated correlations were recognized with *P*<0.05.

**Additional file 3** Critical genera identified by SPD underlying the progression of human gut microbial community along with aging.

**Additional file 4** Supplementary figures. The relative abundance of all the 35 critical genera across different age groups.

## Data Availability

The data we used in this paper was downloaded from previously published paper [10]. The data we generated during data analysis was released as additional files.

## Abbreviations

MST: Minimum spanning tree
OTU: Operational taxonomic unit
PERMANOVA: Permutational analysis of variance
SPD: Sample progression discovery
