# Correction to: Aging progression of human gut microbiota

**DOI:** 10.1186/s12866-021-02200-7

**Published:** 2021-04-28

**Authors:** Congmin Xu, Huaiqiu Zhu, Peng Qiu

**Affiliations:** 1 Department of Biomedical Engineering, College of Engineering, Peking University, Beijing, 100871 China; 2 Department of Biomedical Engineering, Georgia Institute of Technology and Emory University, Atlanta, 30332 USA

**Correction to: BMC Microbiol 19, 236 (2019)**

**https://doi.org/10.1186/s12866-019-1616-2**

Following the publication of the original article [1], we were notified that the supplementary material contains the incorrect links.

The corrected Additional files can be found below:

## Supplementary Information

**Additional file 1: Supplementary Table 1.** Significant genera from PERMANOVA analysis

**Additional file 2: Supplementary Table 2.** Genera correlated with aging with Spearman correlation

**Additional file 3: Supplementary Table 3.** Critical genera revealed by SPD

**Additional file 4: Supplementary Figure 1.** The relative abundance of all the 35 critical genera across different age groups.
