# Supplementary material for: Correction to: Aging progression of human gut microbiota
Source: BMC Microbiol. 2021 Apr 28;21:129. doi: 10.1186/s12866-021-02200-7 (PMC8080400; doi:10.1186/s12866-021-02200-7)
Supplement: Supplementary file 4 — Additional file 4: Supplementary Figure 1. The relative abundance of all the 35 critical genera across different age groups. [file 12866_2021_2200_MOESM4_ESM.pdf]

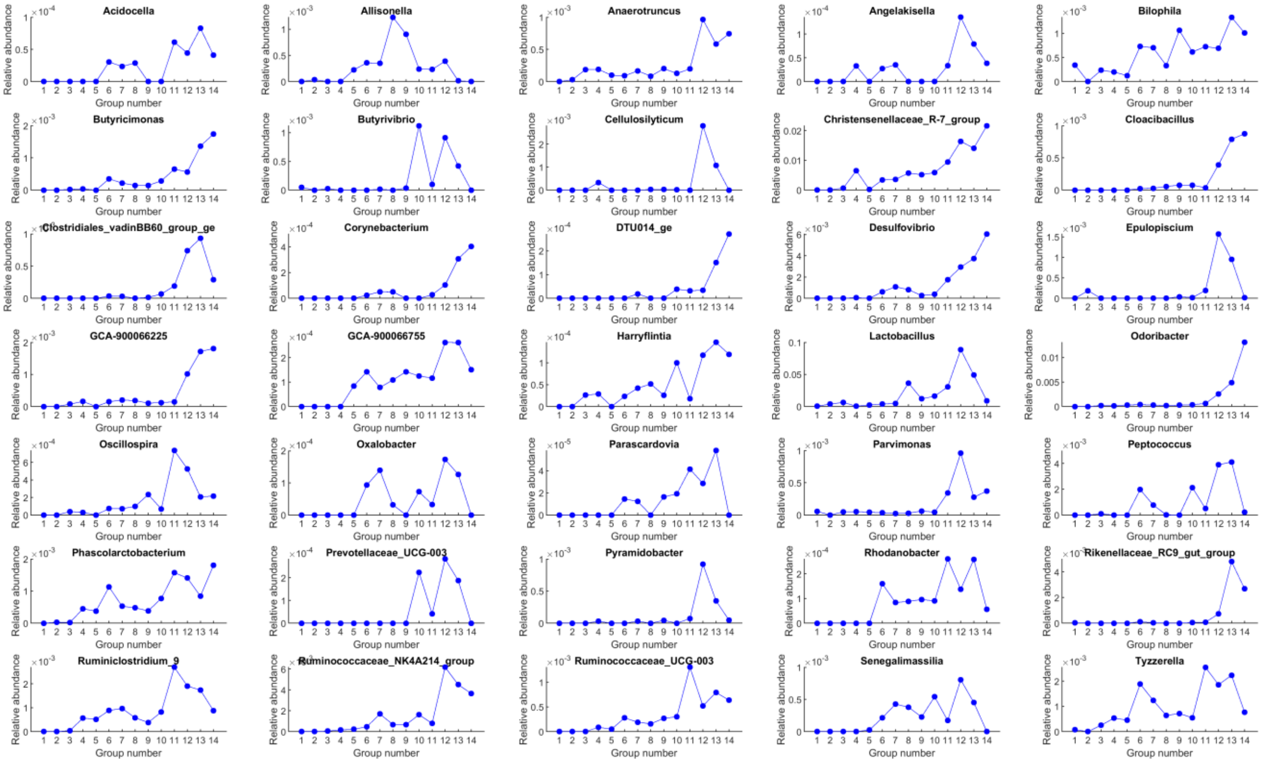

Supplementary figure 1. The relative abundance of all the 35 critical genera across different age groups.

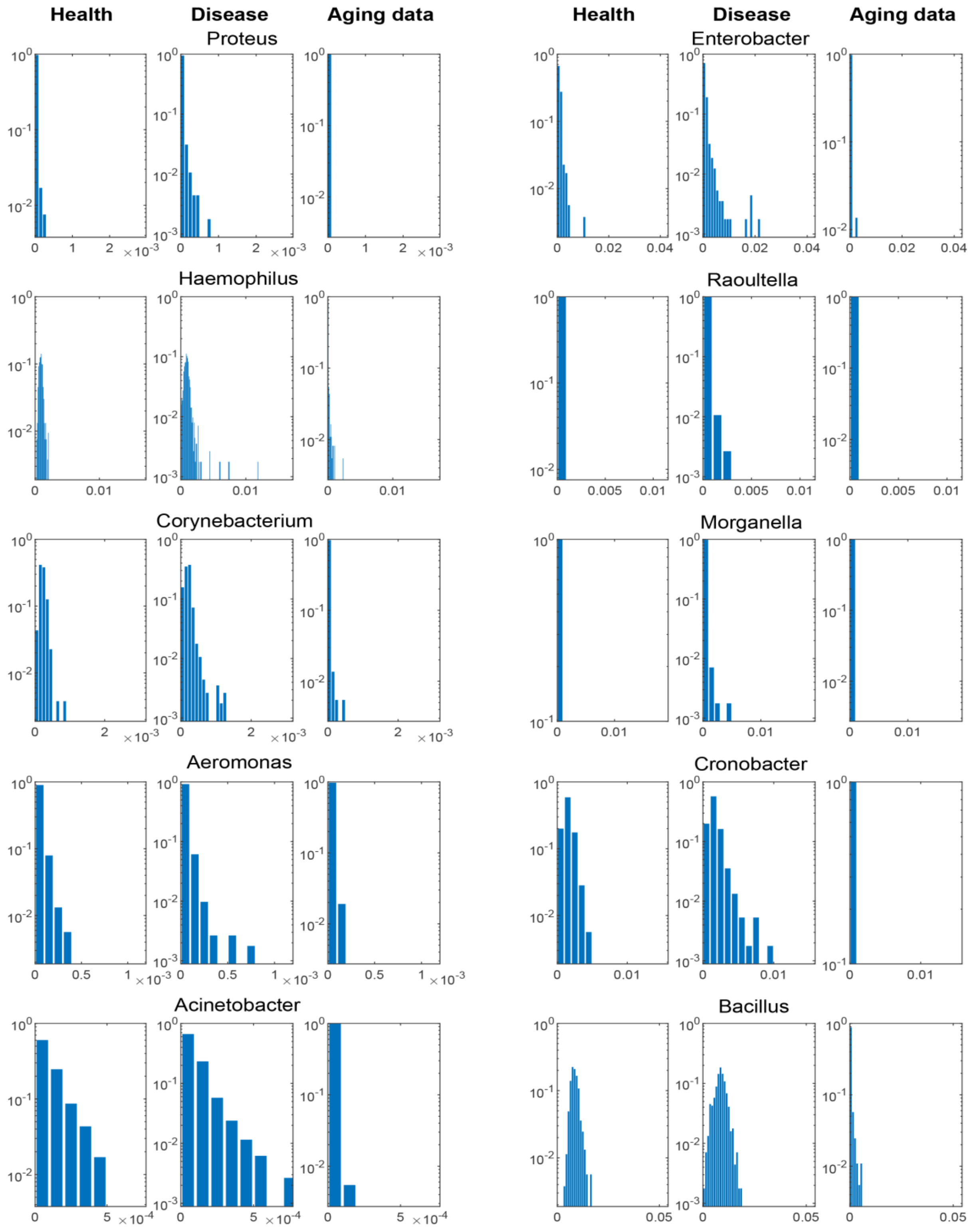

Supplementary figure 2. Frequency histogram of the relative abundance of disease-enriched genera distributed in different kinds of samples in our previous studies and the current dataset. All the value bins along the x-axis are consistent and all the relative abundance values were log transformed before being binned. We could see that the distribution of the samples we included in this paper is more similar to the healthy samples and exhibit lower abundance compared to the disease samples.

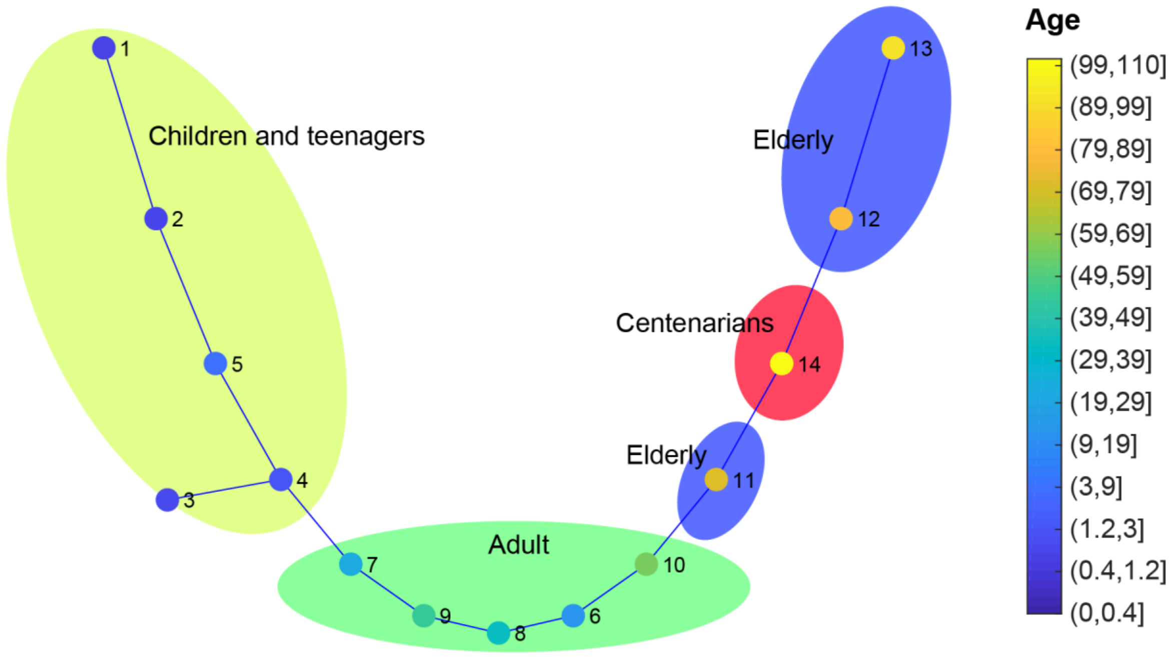

Supplementary figure 3. The minimal spanning tree generated from SPD based on OTUs.

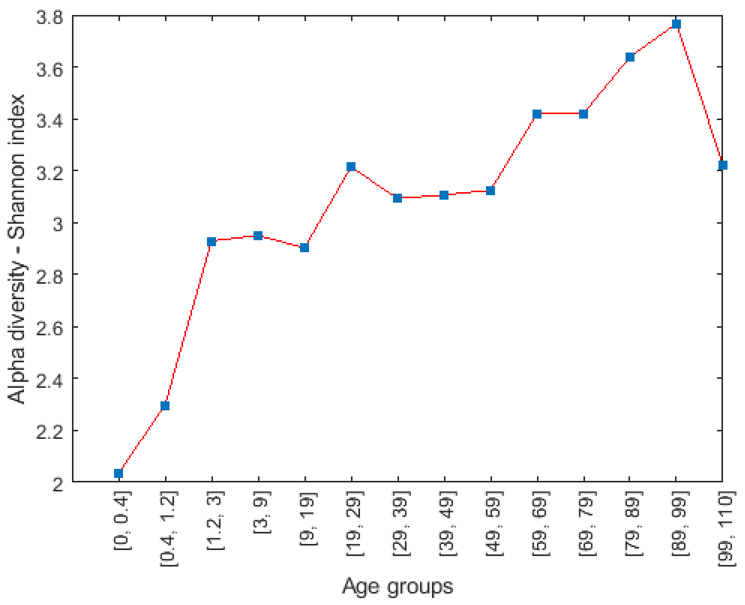

Supplementary figure 4. The alpha diversity of samples in different age groups. Herein, the alpha diversity is quantified by Shannon index. We could see that the alpha diversity truly decreased for the extremely elderly age groups.

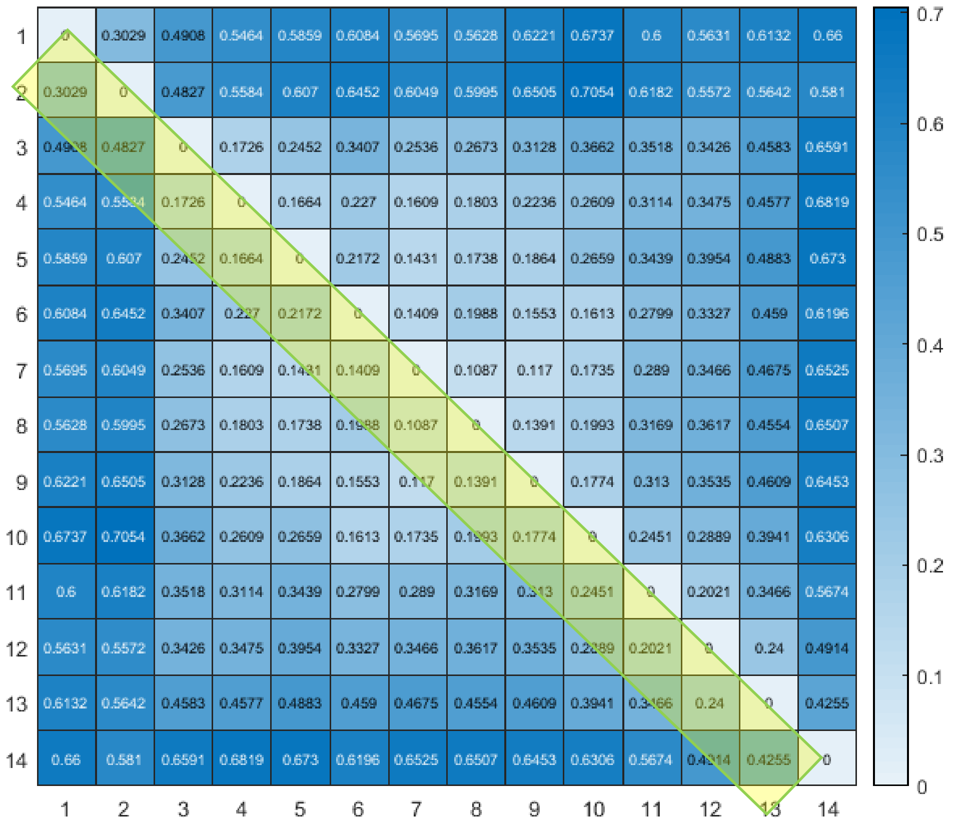

Supplementary figure 5. The beta diversity between different age groups, which is quantified by Bray-Curtis dissimilarity. The values under yellow shadow elucidate the dissimilarity between neighboring age groups.
